# Molecular constraints of sarcopenia in the ageing muscle

**DOI:** 10.3389/fragi.2025.1588014

**Published:** 2025-07-03

**Authors:** S. Damanti, E. Senini, R. De Lorenzo, A. Merolla, S. Santoro, C. Festorazzi, M. Messina, G. Vitali, C. Sciorati, A. A. Manfredi, P. Rovere-Querini

**Affiliations:** ^1^ Internal Medicine Unit, IRCCS San Raffaele Scientific Institute, Milan, Italy; ^2^ Faculty of Medicine and Surgery, Vita-Salute San Raffaele University, Milan, Italy

**Keywords:** sarcopenia, muscle, ageing, molecular mechainsm, constraints

## Abstract

Sarcopenia, the age-related loss of skeletal muscle mass, strength, and function, is driven by a convergence of molecular, cellular, hormonal, nutritional, and neurological alterations. Skeletal muscle comprises multinucleated fibers supported by satellite cells—muscle stem cells essential for repair and regeneration. With age, both the structure and function of these components deteriorate: myonuclei become disorganized, gene expression skews toward catabolic, inflammatory, and fibrotic pathways, and satellite cell numbers and activity decline. Concurrently, mitochondrial dysfunction, impaired proteostasis, and vascular rarefaction limit energy availability and regenerative capacity. Neurodegeneration and age-related muscle fibers denervation further exacerbate muscle loss, particularly affecting fast-twitch fibers, and reduce motor unit integrity. These neural deficits, alongside changes at the neuromuscular junction, contribute to functional decline and diminished contractility. Hormonal changes—including reduced levels of growth hormone, testosterone, and IGF-1—undermine anabolic signaling and promote muscle atrophy. Nutritional factors are also pivotal: anorexia of aging and reduced dietary protein intake lead to suboptimal nutrient availability. Compounding this is anabolic resistance, a hallmark of aging muscle, in which higher levels of dietary protein and amino acids are required to stimulate muscle protein synthesis effectively. Physical inactivity and immobility, often secondary to chronic illness or frailty, further accelerate sarcopenia by promoting disuse atrophy. The molecular constraints of sarcopenia are deeply intertwined with non-molecular mechanisms—such as neuromuscular degeneration, hormonal shifts, inadequate nutrition, and reduced physical activity—creating a complex and self-reinforcing cycle that impairs muscle maintenance and regeneration in the elderly. This review synthesizes current evidence on these interconnected factors, highlighting opportunities for targeted interventions to preserve muscle health across the lifespan.

## 1 Introduction

Skeletal muscle plays a central role in maintaining mobility, metabolic regulation, and overall physiological resilience. Beyond its contractile function, muscle is a dynamic organ involved in thermogenesis, glucose uptake, amino acid storage, and endocrine signaling. As individuals age, the progressive decline in muscle mass, strength, and quality—collectively termed sarcopenia—emerges as a major contributor to frailty, functional impairment, and increased morbidity and mortality. This age-associated muscle degeneration is not merely a consequence of reduced physical activity but results from a complex interplay of molecular, cellular, hormonal, neurological, and nutritional factors.

Understanding the biological mechanisms that underlie sarcopenia is essential for developing effective strategies to preserve muscle health in the elderly. Research over the past decades has revealed a set of molecular constraints that hinder muscle maintenance and regeneration with aging. These include impaired satellite cell function, myonuclear disorganization, chronic inflammation, mitochondrial dysfunction, and disrupted protein homeostasis. These molecular alterations are compounded by systemic changes such as neurodegeneration, hormonal decline, anabolic resistance, and reduced mobility, creating a multifactorial network of degeneration.

In this review, we examine the molecular and cellular changes that constrain muscle adaptation and regeneration in aging. We also explore how these interact with broader systemic factors to accelerate the progression of sarcopenia. Our goal is to provide a comprehensive perspective on the molecular landscape of aging muscle and identify potential targets for therapeutic intervention.

## 2 Muscle architecture and satellite cell niche

Skeletal muscle is a complex and heterogeneous organ primarily composed of multinucleated fibers with different contractile and metabolic functions—slow-twitch/oxidative fibers (type I) and fast-twitch/glycolytic fibers (type II). These fibers are syncytial structures housing hundreds of nuclei (myonuclei), which are usually viewed as post-mitotic. In each fiber, myonuclei are situated peripherally and evenly spaced beneath the sarcolemma (Bruunsgaard et al., 2001; Bruusgaard et al., 2006), while the cytoplasm is primarily filled by longitudinally arranged muscle fibers. The number and spatial arrangement of these myonuclei is vital for muscle fibers function and play a key role in determining the size of mammalian skeletal muscles (Cramer et al., 2020). Each myonucleus controls a specific volume of cytoplasm, known as the myonuclear domain, which helps minimize the need for extensive cytosolic transport of gene and protein products (Bruunsgaard et al., 2001; Blau et al., 1990), ensuring efficient distribution (Ha et al., 1989; Pavlath et al., 1989). The spatial positioning of myonuclei is precisely regulated to maximize their separation (Bruusgaard et al., 2006) without surpassing the functional capacity of each myonucleus (the physiological “ceiling” (Qaisar and Larsson, 2014)), thereby supporting sustainable muscle development throughout life (Cramer et al., 2020).

Myonuclei also function as cellular mechanosensors (Cho et al., 2017; Kirby and Lammerding, 2018), which makes their optimal spatial organization even more crucial. The nuclear envelope and its nuclear lamina physically separate the nuclear genome from the cytoplasm, and together, they mediate the transmission of cytoplasmic mechanical forces to the nuclear interior, thus promoting changes in the arrangement of chromatin and nuclear domains (Maniotis et al., 1997; Lombardi et al., 2011; Guilluy et al., 2014).

Each myonucleus might perform unique functions and express various sets of genes. Specific regions of the muscle fibers exhibit functional specialization, necessitating localized transcripts, such as at the neuromuscular junction (Sanes and Lichtman, 2001; Hippenmeyer et al., 2007; Burden et al., 2018) and muscle-tendon connection sites (Charvet et al., 2012). Consequently, it has been suggested that modest transcriptional heterogeneity among myonuclei arises due to their association with distinct anatomical locations and the influence of stochastic events (Kim et al., 2020; Petrany et al., 2020; Saltin, 1983; Bottinelli et al., 1999; Trappe et al., 2015)

In addition to these multinucleated fibers, muscle tissue contains mononucleated adult myogenic stem cells, known as satellite cells. Other mononucleated cell populations within the muscle include connective tissue cells (e.g., fibroblasts), immune cells (myeloid, lymphoid and mast cells), endothelial cells, pericytes, smooth muscle cells, glial cells (e.g., Schwann cells), and non-myogenic mesenchymal progenitors (e.g., fibro-adipogenic progenitors (FAPs)) (Saltin, 1983; Bottinelli et al., 1999). These various cell types interact with muscle fibers and satellite cells, playing a crucial role in maintaining skeletal muscle homeostasis.

Satellite cells are adult myogenic stem cells (Trappe et al., 2015) situated between the basal lamina and the sarcolemma of skeletal muscle fibers (Giordani et al., 2019). They are small, with minimal cytoplasm and organelles, and possess a single heterochromatic nucleus (Rubenstein et al., 2020). In mature muscles, satellite cells are typically in a resting state; however, they become activated in response to physiological or pathological stimuli such as exercise, mechanical injury, denervation, or muscle dystrophy. Upon activation, these cells re-enter the cell cycle, proliferate, and produce myoblasts. Some of these daughter cells revert to a quiescent state to maintain the satellite cell pool (Dumont et al., 2015; Mauro, 1961; Muir et al., 1965; Schultz et al., 1978; Parise et al., 2008). Myoblasts derived from satellite cells can either repair existing muscle fibers to accommodate muscle turnover or fuse to form new multinucleated fibers, thereby contributing to skeletal muscle adaptability (Lepper et al., 2011; Relaix and Zammit, 2012).

**FIGURE 1:**
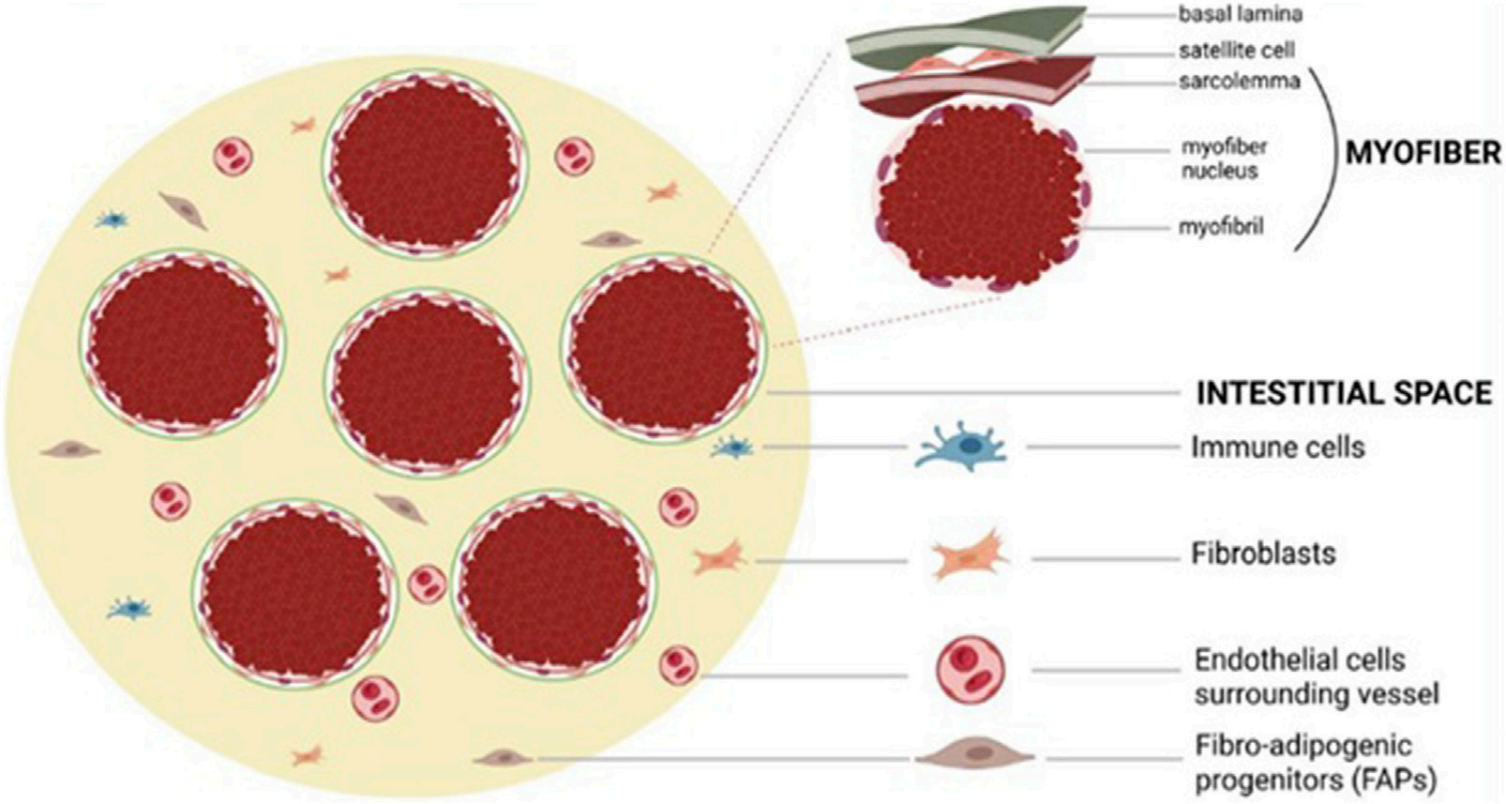
Muscle Fibers and Resident Mononucleated Cells. This illustration depicts the microanatomy of skeletal muscle, highlighting the spatial relationships between multinucleated muscle fibers and key resident mononucleated cell populations. Myonuclei are located peripherally within the muscle fibers, while satellite cells are positioned between the sarcolemma and the basal lamina. The interstitial space contains a variety of cell types, including fibro-adipogenic progenitors (FAPs), immune cells, fibroblasts, and endothelial cells, which interact with muscle fibers and contribute to tissue homeostasis, regeneration, and remodeling. This cellular niche plays a central role in the maintenance of muscle integrity and its response to aging and pathological stressors.

Importantly, satellite cells are heavily influenced by their local microenvironment, known as the satellite cell niche, during activation, proliferation, and differentiation. Various environmental factors, including cytokines, growth factors, free radicals, ion concentrations, and mechanical stimuli, can initiate intracellular signalling pathways that ultimately affect the satellite cell nucleus and regulate gene expression (Yin et al., 2013; Snijders et al., 2015). Figure 1 illustrates muscle fibers and other resident cells populations.

Throughout life, skeletal muscle mass responds to various stimuli, such as growth factors, hormones, external loading, and neural activity (Forcina et al., 2019).

## 3 Muscle ageing and sarcopenia

Skeletal muscle undergoes a series of gradual structural and functional changes with aging—a process commonly referred to as muscle aging. This physiological phenomenon includes reduced regenerative potential, decreased mitochondrial efficiency, and changes in fiber composition, even in otherwise healthy individuals. Moreover, muscle aging is characterized by an upregulation of pathways related to immune response and inflammation, such as the “NF-κB signalling pathway,” “Jak-STAT signalling pathway,” “TNF signalling pathway,” and “Cytokine-cytokine receptor interaction.” Additionally, pathways involved in cell proliferation and growth—including the “p53 signalling pathway,” “Cell cycle,” “Cellular senescence,” and “PI3K-Akt signalling pathway” — also show increased gene activity with age. Conversely, the expression of genes associated with metabolic functions, particularly those involved in “Oxidative phosphorylation,” the “Citrate cycle,” and “Glycolysis,” tends to decline as muscles age (Börsch et al., 2021). While these changes contribute to a general decline in muscle quality, they do not always result in clinically significant impairment.

Aging is marked by a progressive loss of skeletal muscle mass and strength. Typically, lean muscle mass declines from about 50% of total body weight in young adults to approximately 25% in individuals over the age of 80 (Brancaccio and Palacios, 2015). The mechanisms driving this process are complex and multifactorial, involving both intrinsic biological aging and extrinsic lifestyle and health-related factors. However, they are not yet fully understood (Hikida, 2011). These gradual changes in muscle physiology set the stage for sarcopenia—a more severe and pathological form of muscle decline. Sarcopenia, is a clinical syndrome characterized by a more severe and generalized reduction in skeletal muscle mass, strength, and physical performance. It is now recognized as a distinct disease entity (ICD-10-CM code M62.84) and is strongly associated with frailty, falls, disability, and mortality. Sarcopenia reflects a maladaptive or accelerated form of muscle aging in which compensatory mechanisms fail to preserve muscle homeostasis.

While age-related changes in muscle physiology lay the groundwork, sarcopenia results from the interaction of molecular constraints—such as chronic inflammation, satellite cell dysfunction, impaired protein synthesis, and mitochondrial decline—with systemic factors like hormonal insufficiency, physical inactivity, malnutrition, and neuromuscular degeneration.

In addition, one of the key questions in aging research concerns identifying the earliest indicators of the aging process. In humans, two distinct waves of change were identified: an initial phase of metabolic remodelling during young adulthood, followed later by shifts in cellular composition and increased inflammation (Börsch et al., 2021). These observations are consistent with recent findings from a study examining the human plasma proteome across the lifespan, which also reported that changes in specific biological pathways occur in distinct waves during the fourth, seventh, and eighth decades of life (Lehallier et al., 2019).

### 3.1 Cellular senescence

Cellular senescence refers to a permanent halt in the cell cycle that occurs when healthy cells reach their limited ability to replicate, a process known as replicative senescence (Hayflick and Moorhead, 1961). Various stressors can trigger this state, including DNA damage, telomere shortening, oxidative stress, mitochondrial dysfunction, activation of oncogenes, and exposure to chemotherapeutic drugs (van Deursen, 2014). These stimuli cause cell-cycle arrest by activating several pathways, notably the two key regulatory axes: the p53–p21^Cip1 and p16^Ink4a-Rb pathways. Activation of these pathways inhibits cyclin-dependent kinases (CDK2 and CDK4/6), leading to hyperphosphorylation of the retinoblastoma (Rb) protein and ultimately resulting in the cell exiting the cycle (Sharpless and Sherr, 2015; Mankhong et al., 2020).

Previous studies indicated that senescent satellite cells accumulate in the skeletal muscles of aged rodents and elderly humans, as evidenced by increased p16^Ink4a expression and positive senescence-associated β-galactosidase staining (Sousa-Victor et al., 2014; Baker et al., 2008). However, a more recent study did not confirm the presence of p16^Ink4a- or p21^Cip1-positive cells in the skeletal muscles of older individuals (Idda et al., 2020). Anyway, the involvement of p16^Ink4a in sarcopenia has been a particular focus, especially in satellite cells. Sousa-Victor and colleagues demonstrated that silencing p16^Ink4a in aged satellite cells restored their quiescence and muscle regeneration abilities (Sousa-Victor et al., 2014). Similarly, Baker et al. showed that removing p16^Ink4a in RubR1 progeroid mice delayed the onset of sarcopenia (Baker et al., 2011). While the removal of senescent cells has been shown to help prevent sarcopenia (Baker et al., 2011) p21^Cip1 plays a crucial role in completing muscle cell differentiation (Zhang et al., 1999). Additionally, p53 can have a protective effect by slowing the functional decline of skeletal muscle cells through a p21^Cip1-dependent mechanism that involves the suppression of p16^Ink4a (Baker et al., 2013). Senescent cells exhibit a distinctive secretory profile known as the senescence-associated secretory phenotype (SASP), which includes a variety of cytokines, proteases, chemokines, growth factors, and extracellular vesicles. Notably, the effects of the SASP can be either beneficial or harmful, depending on the specific makeup of different cell types and the nature of the stressors that trigger senescence (Coppé et al., 2008). The accumulation of senescent cells promotes the production of SASP, which contributes to the chronic, low-grade inflammation known as “inflammaging.” Inflammatory signals activate IκB kinase (IKK), which phosphorylates IκB—an inhibitor that normally binds nuclear factor kappa B (NF-κB) and keeps it inactive in the cytoplasm. Phosphorylation of IκB targets it for degradation by the proteasome, thereby releasing NF-κB. Once freed, NF-κB translocates into the nucleus, where it stimulates the expression of pro-inflammatory genes and upregulates muscle ring finger 1 (MuRF1), a key factor promoting muscle protein degradation (Mankhong et al., 2020).

Growing evidence shows that serum levels of tumor necrosis factor (TNF)-α, interleukin (IL)-6, and C-reactive protein (CRP) are elevated in individuals with sarcopenia, typically reaching levels 2–4 times higher than those observed in younger controls. A cross-sectional study by Bain et al. found that older individuals with sarcopenia had higher serum levels of IL-6 and TNF-α compared to those without sarcopenia (Bian et al., 2017). Similarly, Marzetti et al. reported increased levels of CRP, P-selectin, and interferon-induced protein 10 in individuals with physical frailty and sarcopenia (Marzetti et al., 2019).

### 3.2 Age-related structural and functional alterations in myonuclei

Skeletal muscle fibers are a syncytia containing hundreds of nuclei (myonuclei), which are generally considered post-mitotic. However, Boro et al. (2022) observed DNA synthesis in the tibialis anterior myonuclei of mice, suggesting that adult muscle fibers are not completely post-mitotic. Each myonucleus may perform distinct functions and express different gene sets, particularly at functionally specialized regions like the neuromuscular junction and muscle–tendon junction. Modest transcriptional heterogeneity among myonuclei is believed to arise from anatomical positioning and stochastic events (Kim et al., 2020; Bagley et al., 2023; Williams et al., 2022; Cisterna and Malatesta, 2024).

Myonuclei are peripherally located beneath the sarcolemma. Their spatial distribution is crucial to maintain functional domains and efficient intracellular communication. Recent studies reported a decreased count of myonuclei aged muscles (Kim et al., 2020; Bagley et al., 2023; Williams et al., 2022; Cisterna and Malatesta, 2024; Kirby and Dupont-Versteegden, 2022; Lai et al., 2024). Moreover, alterations in structure and function of myonucle indicating disrupted RNA processing have also been reported (Malatesta et al., 2009).

Aging is associated with structural and spatial alterations in myonuclei. In aged muscle fibers, myonuclei are often irregular in shape, deviating from the elliptical forms typical of young fibers and exhibiting nuclear envelope indentations (Brooks et al., 2009). These changes have been linked to age-related disruptions in the surrounding microtubule network (Brooks et al., 2009). Loss or repositioning of myonuclei can compromise the size and function of the myonuclear domain, particularly in fast-twitch Type II fibers where a reduction in domain size has been observed. Conversely, slow-twitch Type I fibers may display enlarged domains with fewer myonuclei. Such reorganization may hinder protein turnover and impair contractile protein distribution, contributing to muscle weakness and sarcopenia (Cisterna and Malatesta, 2024; Azevedo and Baylies, 2020).

Myonuclei also act as mechanosensors (Kirby and Lammerding, 2018; Lai et al., 2024; Azevedo and Baylies, 2020; Folker and Baylies, 2013), and their laminand nuclear pore complexes transmit mechanical cues to the genome. In aged quadriceps muscle, Iyer et al. (2021) reported reduced expression of lamin-β1, fewer nuclear pores, and altered nucleoporin expression. These impairments likely compromise mechanosignalling, increasing nuclear permeability and promoting sarcopenia.

Alterations in nuclear shape and chromatin architecture profoundly affect transcriptional regulation. In aged muscle, myonuclei tend to be smaller, with a higher degree of chromatin condensation, reduced levels of RNA polymerase II and splicing factors, and mislocalization of key RNA processing machinery (Cisterna and Malatesta, 2024; Malatesta et al., 2009). These transcriptional deficits are further compounded by epigenetic dysregulation, notably hypermethylation of gene regions, which interferes with normal gene expression patterns in aging muscle (Turner et al., 2020). Encouragingly, physical exercise has been shown to partially reverse these deficits, restoring transcriptional activity and reducing hypermethylation in key regulatory regions (Murach et al., 2021).

Recent findings underscore fiber-type specific effects of aging on myonuclei. Type II fibers, more vulnerable to atrophy, exhibit more pronounced transcriptional impairments. These include upregulated markers of proteolysis and oxidative stress, and reduced contractile protein synthesis—features linked to anabolic resistance and degeneration. These deficits are further exacerbated by impaired mechano-transduction and growth factor responsiveness, undermining muscle adaptability and contributing to sarcopenia (Brooks et al., 2009).

### 3.3 Reduction of satellite cells

Aging profoundly affects satellite cells—postnatal muscle stem cells located between the basal lamina and the sarcolemma of skeletal muscle fibers (Fu et al., 2015). These cells are small, with limited cytoplasm and a single heterochromatic nucleus, and typically remain in a quiescent state in adult muscle. Upon stimulation—such as through exercise, injury, or disease—satellite cells become activated and re-enter the cell cycle. They divide asymmetrically to give rise to two daughter cells: one cell initiates the myogenic program, becoming a myoblast that will contribute to muscle fiber repair or regeneration; the other returns to a quiescent state to maintain the stem cell pool and ensure long-term regenerative capacity (Byun et al., 2024; Karami et al., 2025).

Satellite cell fate is tightly controlled by the surrounding niche—a specialized microenvironment rich in signaling molecules and mechanical stimuli. Factors such as cytokines, growth factors, oxidative stress, ion fluxes, and mechanical loading regulate gene expression and signaling pathways that determine satellite cell activation, proliferation, and differentiation (Loreti and Sacco, 2022).

In aging muscle, satellite cells experience both quantitative and functional impairments. While the degree of cell loss may vary by species and fiber type, evidence consistently shows a reduced satellite cell pool and diminished responsiveness to activation signals. These changes are associated with altered molecular signaling, including impaired Notch pathway activation due to reduced Delta ligand expression (Conboy and Rando, 2002). Additionally, chronic inflammation, elevated cellular stress, and altered transcriptional regulation further compromise their regenerative capacity (Careccia et al., 2023).

Nonetheless, physical exercise remains a potent modulator of satellite cell activity, even in older individuals. Exercise has been shown to partially restore satellite cell responsiveness and reinitiate pre-mRNA processing, although the regenerative response is blunted compared to that in younger muscle (Snijders et al., 2019).

Hormonal regulation also plays a key role. Androgens, particularly testosterone, support satellite cell activation and proliferation, and satellite cells express androgen receptors (Barsky and Monks, 2025; Fu et al., 2024; Kraemer et al., 2020). Aging is associated with a reduction in both androgen levels and receptor expression. Di Donato et al. (2023) found lower androgen receptor expression in biopsies of gluteus medius and vastus medialis muscles from older adults, and demonstrated androgen-induced nuclear translocation of the receptor in murine myoblasts. These findings suggest that reduced androgen signaling contributes to satellite cell dysfunction and impaired muscle regeneration in aging.

### 3.4 Altered microvascular function

Proper vascular supply is essential for nutrient delivery and waste clearance in muscle tissue. Aging is marked by endothelial dysfunction, atherosclerosis, and heightened alpha-adrenergic vasoconstriction, all of which reduce muscle blood flow at rest and during exercise (Richards et al., 2014). These vascular changes hinder the efficient delivery of oxygen and nutrients to muscles, accelerating the onset of sarcopenia. Additionally, the reduction in capillary density, often a consequence of sedentary lifestyles prevalent among older adults, exacerbates muscle performance decline (Distefano and Goodpaster, 2018). Finally, the downregulation of genes associated with cell junction assembly and transmembrane transport, coupled with heightened pro-inflammatory and chemoattractant signalling, leads to increased immune cell activity and amplified inflammatory responses. This cascade promotes greater infiltration of mast cells, lipid-associated macrophages, and monocytes into aging muscle tissue, significantly contributing to the progressive decline in muscle health (Shen et al., 2023; Zhou et al., 2025).

### 3.5 Fibrotic and adipogenic shifts

With aging, there is a notable rise in fibroblast-like cells and adipocytes, while the number of Fibro-Adipogenic Progenitors (FAPs) decreases. Older FAP subtypes show an aging-related signature, characterized by the downregulation of growth factor pathways and the upregulation of profibrotic and pro-inflammatory pathways. These molecular alterations reflect a shift toward a more fibrotic and inflammatory environment in aging tissues, contributing to tissue dysfunction and impaired muscle regeneration (Kirby and Dupont-Versteegden, 2022; Lukjanenko et al., 2019). Aging is also associated with ectopic fat accumulation within muscles. This, along with excessive extracellular matrix deposition—particularly collagen—leads to increased muscle stiffness, impaired muscle regeneration, and reduced strength and performance, contributing to the progressive decline in muscle mass and function. A comparative analysis across different age groups (15–46 years, 74–82 years, and ≥84 years) revealed that pro-inflammatory pathways, such as IL-6/AP-1, are most active in older individuals (74–82 years), while profibrotic pathways, particularly TGFβ signalling, are most prominent in the oldest group (those 84 years and older) (Kirby and Dupont-Versteegden, 2022).

### 3.6 Disrupted protein homeostasis

Protein homeostasis—or proteostasis—refers to the dynamic balance between protein synthesis and degradation, which is essential for maintaining muscle mass, structure, and function. In skeletal muscle, this balance is tightly regulated by hormonal signals, nutrient availability, mechanical loading, and intracellular pathways. With aging, this equilibrium becomes increasingly impaired, leading to reduced muscle protein synthesis, heightened protein degradation, and accumulation of damaged or misfolded proteins.

Disrupted proteostasis is now widely recognized as a central driver of sarcopenia because skeletal muscle is a protein-rich tissue with high metabolic demands. Unlike other mechanisms that may operate locally or episodically (e.g., denervation, inflammation), impaired proteostasis affects virtually all muscle cells continuously and directly compromises their structural integrity and function. Moreover, it integrates signals from systemic factors (e.g., hormones, inflammation, nutrition) and intrinsic cellular machinery (e.g., mTOR, autophagy, ubiquitin-proteasome system), serving as a converging point for many aging-related stressors. For this reason, the breakdown of protein homeostasis is considered one of the primary molecular constraints that initiate and sustain muscle degeneration during aging. The following subsections detail how aging impairs both sides of the proteostasis equation—synthesis and degradation.

#### 3.6.1 Impaired protein synthesis

Protein synthesis in muscle cells is influenced by several factors, including: (i) amino acids, particularly branched-chain amino acids like leucine; (ii) physical exercise; (iii) insulin and insulin-like growth factor-1 (IGF-1); and (iv) various hormones which act on the mammalian target of rapamycin (mTOR) pathway (Yoon, 2017; Rooyackers and Nair, 1997; Smith et al., 2012). mTOR is a serine/threonine kinase encoded by the *MTOR* gene, located at 1p36.22. Structurally, its C-terminal houses the catalytic (kinase) domain, while its N-terminal contains domains facilitating protein-protein interactions necessary for assembling TOR into two distinct complexes: TOR complex 1 (TORC1) and TOR complex 2 (TORC2). Insulin-like growth factor-1 (IGF1) and physical activity initiate a signalling cascade that activate class 1 phosphatidylinositol 3-kinase PI3K which phosphorylate AKT and then activate TORC1. In contrast, the class 3 PI3K pathway activates TORC1 in response to amino acids, particularly L-leucine without AKT phosphorylation. Once activated, TORC1 phosphorylates two key effectors involved in mRNA translation: eukaryotic initiation factor 4E-binding protein-1 (4EBP1) and ribosomal protein p70 S6 kinase-1 (p70^S6K1^) (Ragupathi et al., 2024). Older individuals exhibit a reduced TORC1 signalling and ability to stimulate skeletal muscle protein synthesis in response to physical exercise compared to younger individuals (Yoon, 2017). Aging has been linked also to an impaired activation of TORC1 in response to amino, a condition notably known as anabolic resistance (Tezze et al., 2023).

##### 3.6.1.1 Influence of hormones on protein synthesis

A decline in anabolic hormones may play a role in accompanying age-related musculoskeletal impairments. In older adults, reduced levels of sex steroids, growth hormone (GH), and insulin-like growth factor-1 (IGF-1) are closely linked to the deterioration of muscle mass and function (Barsky and Monks, 2025; Kraemer et al., 2020; Huang and Wang, 2021). Testosterone, a powerful anabolic hormone, enhances muscle protein synthesis and supports muscular regeneration, while estrogens offer protective benefits to skeletal muscle by mitigating inflammation. Similarly, GH and IGF-1 stimulate protein synthesis and reduce protein degradation (Kraemer et al., 2020). On the other hand, the reduction in muscle mass in older individuals, determine a resistance to the anabolic effects of insulin. Notably the degrees of insulin resistance across glucose, protein, and lipid metabolism vary in older individuals. For instance, many older adults remain insulin-sensitive for glucose metabolism but exhibit resistance when it comes to protein synthesis. Adding to this complexity, muscles with different fiber type compositions display distinct sensitivities to insulin in regulating both glucose and protein metabolism (Rasmussen et al., 2006).

#### 3.6.2 Impaired proteolytic and autophagic pathways

The ongoing breakdown of faulty proteins and organelles, coupled with the production of new proteins, enables muscles to preserve their functionality and adjust to various stimuli.

##### 3.6.2.1 Ubiquitin-proteasome pathway

Most intracellular proteolysis occurs through the ubiquitin-proteasome pathway (UPP), with additional contributions from the autophagy-lysosomal pathway. The UPP involves a 26S proteasome complex composed of 19S and 20S subunits. Ubiquitin is conjugated to target proteins through a tagging process mediated by three enzyme families (E1, E2, and E3), marking these proteins for degradation (Shang and Taylor, 2011). Upregulation of this pathway is a hallmark of muscle atrophy, with increased expression of key E3 ubiquitin ligases, atrogin-1 (MAFbx/FBXO32) and muscle ring finger protein-1 (MuRF1/TRIM63) (Bodine and Baehr, 2014).

The expression of atrogin-1 and MuRF1 is regulated by the phosphorylation status of forkhead box (FOXO) proteins. Under normal conditions, FOXO proteins are phosphorylated by Akt, which sequesters them in the cytoplasm, preventing their nuclear translocation and transcriptional activation of atrogin-1 and MuRF1 (Kim et al., 2021).

**FIGURE 2:**
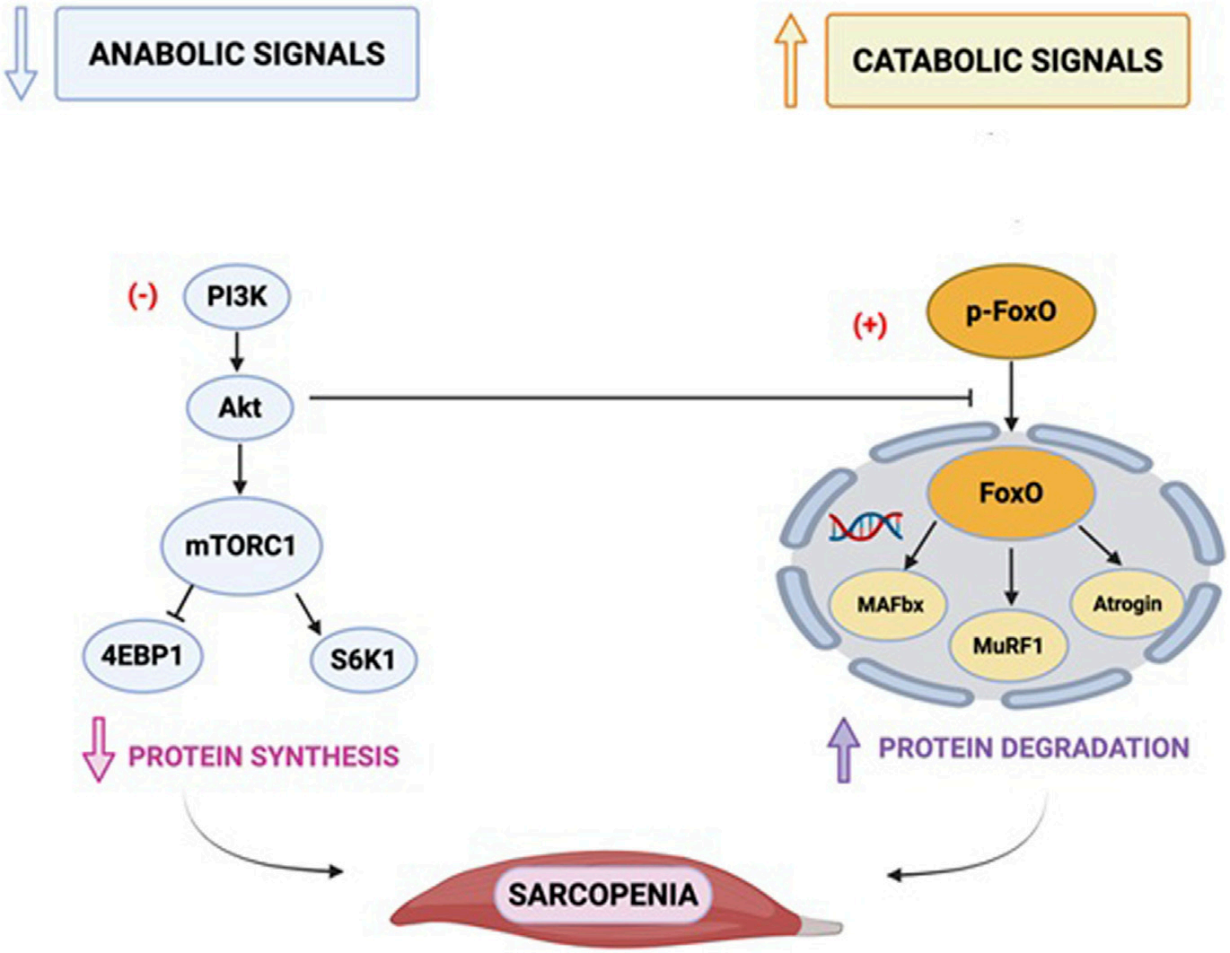
Main molecular mechanisms of protein synthesis and breakdown. PI3: phosphatidylinositol trisphosphate; PDK1: phosphoinositide-dependent kinase-1; Akt: a serine/threonine-protein kinase; TSC: tuberous sclerosis complex; mTOR: mammalian target of rapamycin; FOXO: belongs to the O subclass of the forkhead family of transcription factors which are characterized by a distinct fork head DNA-binding domain. This transcription factor has the ability to be inhibited and translocated out of the nucleus on phosphorylation by proteins such as Akt/PKB in the PI3K signalling pathway; MAFbx: muscle atrophy F-Box/atrogin-1; MuRF1: muscle-specific RING-finger protein 1; 4EBP1: eukaryotic initiation factor 4E-binding protein-1 (4EBP1); p70^S6K1^: ribosomal protein p70 S6 kinase-1. This figure depicts the key signalling pathways involved in muscle atrophy, focusing on the mammalian target of rapamycin (mTOR).

Figure 2 illustrates the main molecular mechanisms underpinning muscle protein synthesis and breakdown.

The active form of vitamin D, 1,25(OH)_2_D_3_, and its receptor (VDR) play key roles in regulating proteolysis in skeletal muscle. 1,25(OH)_2_D_3_ can inhibit the expression of atrogin-1 and MuRF1 while increasing FOXO1 levels (Hayakawa et al., 2015). The widespread deficiency of vitamin D among older adults has significant negative implications for muscle health. This deficiency not only contributes to increased protein breakdown, impairing muscle repair and maintenance. Furthermore, low vitamin D levels may exacerbate inflammation and diminish the effectiveness of anabolic signals necessary for muscle growth and preservation, compounding its detrimental effects on overall musculoskeletal health (Ruggiero et al., 2024).

##### 3.6.2.2 Autophagy

Autophagy is a lysosome-driven degradation mechanism that primarily targets malfunctioning proteins and organelles by breaking them down to recycle impaired cellular components. It involves the creation of a double-membrane vesicle known as an autophagosome, which encapsulates cytosolic material. The autophagosome then merges with a lysosome to form an autolysosome, where enclosed organelles and proteins are broken down (Liu et al., 2023). It is well recognized that autophagy plays a vital role in eliminating protein aggregates and damaged mitochondria. However, ageing impairs the efficacy of autophagy (Lim et al., 2024). It has been demonstrated that, mice lacking autophagy exhibit several age-related traits, including reduced muscle mass and quality (Li P. et al., 2021; Masiero et al., 2009). Moreover, the inadequate clearance of damaged mitochondria through autophagy can lead to elevated reactive oxygen species (ROS) production, which in turn increases protein carbonylation and associated damage, further aggravating muscle impairment (Wang et al., 2023).

### 3.7 Muscle fibers denervation

The integrity of neuromuscular connections is essential for maintaining muscle mass and function. With aging, progressive denervation contributes significantly to muscle fiber atrophy and the onset of sarcopenia (Sayer et al., 2024). Aging is characterized by a reduction in the number of motor neurons and large-diameter axons. Motor neuron loss begins around the age of 50, causing temporary denervation of muscle fibers. This is mitigated by compensatory mechanisms, such as collateral reinnervation by nearby axon terminals. During this temporary denervation, the fibers cannot contract but maintain their contractile machinery. This may help explain why muscle strength and power decline more significantly than muscle mass with aging, although other factors have also been suggested. In later life, motor neuron loss accelerates, and reinnervation eventually becomes insufficient, resulting in permanent muscle fiber denervation (Soendenbroe et al., 2021; You and Chen, 2021). Moreover, aging has been associated with morphological and biochemical changes at the neuromuscular junction (NMJ) in both humans and rodents, which either contribute to or result from NMJ destabilization (Khosa et al., 2019). Motor neurons vary in characteristics such as cell body size, activation threshold, fatigue resistance, tension generation, and transmission speed. Alpha motor neuron cell bodies are located in the ventral horn of the spinal cord and extend myelinated axons to the periphery, where each motor neuron forms multiple specialized synapses with skeletal muscle fibers. These synapses, known as neuromuscular junctions (NMJs), play a critical role in muscle function (Hughes et al., 2006). Notably, the properties of a motor neuron align with those of the muscle fibers it innervates, forming what is known as a motor unit, where all fibers within the unit are of the same type (Hughes et al., 2006). The NMJs are situated at a specialized region of the sarcolemma known as the endplate. In mammalian muscles, a healthy endplate typically exhibits a “pretzel-like” shape formed by the branching terminals of the motor neuron. At the ends of these branches are presynaptic boutons—enlarged structures filled with synaptic vesicles containing the neurotransmitter acetylcholine (ACh) (Hughes et al., 2006). These boutons are precisely aligned with post-synaptic indentations in the sarcolemma called junctional folds. These folds are densely packed with acetylcholine receptors (AChRs), which are essential for synaptic transmission (Liu et al., 2008). Surrounding the junctional folds are perisynaptic Schwann cells (PSCs), specialized glial cells that support and regulate the structure and function of the NMJ (Hughes et al., 2006). When ACh is released into the synaptic cleft, it binds to AChRs on the post-synaptic membrane, generating an endplate potential (EPP). This local depolarization initiates an action potential that propagates across the muscle fiber, ultimately causing muscle contraction and force generation. A healthy neuromuscular connection is crucial for the survival of muscle fibers, as fibers deprived of neural input (denervated) progressively atrophy and eventually die. Consequently, denervation significantly contributes to muscle weakness and frailty in aging (Bao et al., 2020). Reinnervation of denervated muscle fibers can lead to an increase in the size of smaller motor units. This aligns with observations in older adults, who tend to have fewer but larger motor units, up to a certain limit (Piasecki et al., 2016). Fast-twitch type II muscle fibers are more likely to lose their nerve supply, and when they are reinnervated by slow motor neurons, they transform into slower Type I fibers (Gonzalez-Freire et al., 2014).

The neural contribution to muscle wasting appears to be an early event in the onset of sarcopenia and seems to be a significant determinant of reduction of muscle quality (Padilla et al., 2021). However, this process may also be reciprocal, with inactivity potentially contributing to denervation. In addition to anterograde signalling, there is evidence for retrograde signalling, where molecular signals are transported from the presynaptic terminal along the axon back to the cell body in the spinal cord. These signals can be taken up by motor neurons at the NMJ, providing a pathway from the periphery to the central nervous system. A notable example of physiological retrograde signalling is the study by Chakkalakal et al. (2010), which demonstrated that overexpression of PGC1a in muscle fibers, promoting a slow phenotype, induced a corresponding slow phenotype in motor neurons. Moreover, in cases of malnutrition of muscle inactivity, which directly impact on muscle health, an increase in negative retrograde signalling could theoretically lead to muscle fiber denervation, regardless of motor neuron condition. Thus, bidirectional signalling, from nerve to muscle and muscle to nerve, plays a crucial role in motor neuron survival and the maintenance of NMJs.

### 3.8 Mitochondrial dysfunction

Mitochondrial morphological and functional alterations precede the reduction of muscle function and mass typical of the ageing process (Ferri et al., 2020). Mitochondria are organelles critical for maintaining muscle mass and function by providing the energy necessary for movement and metabolic activities via oxidative phosphorylation (OXPHOS). Although mitochondria are primarily recognized for ATP production, they also play crucial roles in apoptosis, cellular metabolic and redox signalling, and calcium homeostasis, collectively linking them to the aging process. Subsarcolemmal mitochondria, characterized by greater interconnectivity compared to intermyofibrillar mitochondria, play a key role in gene expression and the regulation of reactive oxygen species (ROS) levels. In contrast, intermyofibrillar mitochondria are primarily dedicated to supporting oxidative phosphorylation (OXPHOS) and calcium homeostasis. Mitochondrial volume varies across muscle fiber types, with type II (fast-twitch) fibers containing smaller mitochondria than type I (slow-twitch) fibers. To sustain the high energy demands of skeletal muscle, mitochondria rely on well-regulated quality control mechanisms, including oxidant-scavenging systems, mitochondrial DNA maintenance, calcium homeostasis, protein repair and degradation, autophagy and mitodynamics (Spinelli and Haigis, 2018).

#### 3.8.1 Mithocondrial dynamics

Mitochondrial dynamics involve two key processes: fission, facilitated by proteins like dynamin-related protein 1 (DRP1), and fusion, mediated by proteins such as optic atrophy protein 1 (OPA1) and mitofusins 1 and 2 (MFN1 and MFN2) Disruptions in either process can impair mitochondrial function, leading to dysfunction and potential pathological conditions. Age-related declines in OPA1 have been associated with decreased skeletal muscle mass (Tezze et al., 2017), while reductions in MFN2 have been linked to metabolic changes and the development of sarcopenia (Sebastián et al., 2016). Indeed, MFN2 is also important in the regulation of mitochondrial cristae ultrastructure and energy production. Knocking out MFN2 leads to a decrease in the number, volume, and surface area of cristae, which implies a diminished oxidative capacity. Previous research in skeletal muscle has also demonstrated that deleting MFN2 leads to reduced activity of electron transport chain complex I and cause mitochondrial swelling, driven by osmotic imbalances (Zanfardino et al., 2025).

#### 3.8.2 MICOS complex

Aging appears to be also linked to lipid-driven changes in membrane viscosity, which influence the Mitochondrial Contact Site and Cristae Organizing System (MICOS) complex. The MICOS is a protein complex essential for maintaining the structure and organization of mitochondrial cristae and for facilitating contact sites between the inner and outer mitochondrial membranes. These contacts are critical for mitochondrial function and cellular energy production. Thus, alterations in the MICOS complex impact on mitochondrial structure, such as the loss of cristae morphology, as well as overall function (Vue et al., 2024). Indeed, in a murine model of aging, a reduction in both the number and quality of mitochondrial cristae has been showed (Vue et al., 2023).

#### 3.8.3 ATP production

ATP production during aging is further impacted by a decline in Carnitine Acyltransferase levels, an enzyme essential for transporting acyl groups from the cytosol into the mitochondrial matrix to facilitate beta-oxidation (Noland et al., 2009).

#### 3.8.4 Mitochondria and calcium

Mitochondrial calcium (Ca^2+^) uptake is critical for maintaining intrinsic mitochondrial functions, with Ca^2+^ playing a central role in regulating metabolic activity. The Mitochondrial Calcium Uniporter (MCU) and Mitochondria-ER Contact Sites (MERCs) fulfil distinct yet interconnected roles in mitochondrial function and calcium homeostasis. MERCs, which are physical contact points between the mitochondrial outer membrane and the endoplasmic reticulum (ER), facilitate the transfer of calcium, lipids, and other signalling molecules between the two organelles. This process is essential for calcium transfer from the ER to the mitochondria and relies on tethering proteins, such as MFN2, to maintain the structural and functional coupling between the ER and mitochondria. The MCU, a calcium channel located in the inner mitochondrial membrane, mediates the direct uptake of Ca^2+^ into the mitochondrial matrix. This Ca^2+^ influx is vital for regulating key metabolic processes, including the tricarboxylic acid (TCA) cycle, oxidative phosphorylation, and ATP production (Mishra et al., 2017).

The increased expression of MERC proteins (Grp75, Ip3r3, and Vdac3) in human skeletal muscle during aging confirms a compromise in mitochondrial dynamics and structural integrity (Scudese et al., 2024). Excessive expression of MERC proteins and excessive activation of the MCU can lead to increased calcium transfer into mitochondria, causing oxidative stress and mitochondrial dysfunction. This dysfunction may reduce the mitochondria’s ability to maintain their normal dynamics, such as fusion and fission processes, compromising mitochondrial structural integrity. Consequently, these alterations may contribute to the reduction in muscle mass and function observed during aging. Moreover, Ca^2+^ overload can trigger the opening of the mitochondrial permeability transition pore (mPTP) and potentially cause cell death (Abramov and Duchen, 2011). On the other hand, the increase in MERC proteins detected during ageing could also reflect a compensatory attempt by the cell to maintain calcium homeostasis and cellular signalling in response to aging-associated stress.

#### 3.8.5 Mitochondria and oxidative stress

Mitochondria are a primary source of oxidants, and aging is closely linked to impaired mitochondrial respiratory function and elevated production of reactive oxygen species (ROS), although the severity of these changes is largely influenced by an individual’s level of physical activity (Hepple, 2016). Oxidative stress establishes a vicious cycle in which mitochondrial dysfunction further amplifies ROS generation, thereby accelerating cellular senescence.

#### 3.8.6 Mitochondria swelling

Ca^2+^ overload or oxidative stress can induce mitochondrial swelling, leading to an increase in mitochondrial volume, as observed in aged samples. Notably, this volume increase occurs without corresponding changes in mitochondrial surface area or perimeter, which remain unchanged. Swelling disrupts cristae structure, impairing ATP production. Moreover, it is coupled with a loss of membrane potential, which typically precedes the opening of the mitochondrial mPTP, a process that can ultimately result in cell death. Indeed, swelling often precedes apoptosis (Scudese et al., 2024).

#### 3.8.7 Mitochondrial DNA

The replication, deletion, and mutation rates of mitochondrial DNA (mtDNA) increase with age, leading to significant impacts on cellular metabolism and mitochondrial function. Elevated circulating levels of mtDNA have been linked to sarcopenia. This association underscores the critical role of mitochondrial health in maintaining muscle integrity and highlights mtDNA as a potential biomarker and contributor to age-related muscle degeneration (Fan et al., 2022)

#### 3.8.8 Mitochondrial extracellular vescicles

In recent years, extracellular vesicles (EVs) have emerged as important mediators of intercellular communication, carrying a variety of bioactive molecules, including mitochondrial components. The release of mitochondrial components through extracellular vesicles (EVs) has recently been recognized as an important mechanism of cellular quality control and intercellular communication, particularly in the context of aging. Mitochondria-derived EVs play a pivotal role in regulating inflammatory pathways, maintaining tissue homeostasis, and mitigating mitochondrial dysfunction. However, increasing evidence suggests that impairments in the biogenesis and secretion of these vesicles may contribute to the development of chronic low-grade inflammation, or “inflammaging,” and age-associated conditions such as sarcopenia. Picca et al. reported that the amount of mitochondrial components in secreted extracellular vesicles (EVs) was lower in sarcopenic elderly individuals compared to non-sarcopenic elderly controls, while the overall serum levels of EVs were higher in sarcopenic patients []. Moreover, mitochondria-derived EVs can carry danger-associated molecular patterns (DAMPs) that can activate sterile inflammatory pathways, such as the Toll-like receptor system and the NLRP3 inflammasome. Thus, mitochondrial dysfunction in aging skeletal muscle may drive “inflammaging,” contributing to the development of sarcopenia (Picca et al., 2020). In this regard, mitochondrial EVs are not only emerging as biomarkers of cellular health and aging but also as potential therapeutic targets aimed at preserving muscle function.

### 3.9 Gut microbiota

The gut microbiota refers to the diverse community of microorganisms residing in the human gastrointestinal tract, including bacteria, fungi, viruses, and other microbes. Among these, bacteria constitute the predominant group. Key bacterial taxa commonly found in the gut include Bacteroidetes, Firmicutes, Proteobacteria, and Actinobacteria. In addition to these major groups, a variety of other microorganisms, such as anaerobic species and members of the Anaerococcus genus, can also be present. The intestinal tract also harbors several types of viruses, including haloviruses and bacteriophages.

The composition of the gut microbiota is influenced by numerous factors such as diet, environmental exposures, age, physiological conditions, and genetic background. As a result, significant variability in microbiota composition exists between individuals (Gao et al., 2024). Alterations in the gut microbiota and its metabolites may play a role in the distinct clinical complexities observed in frail older adults (Casati et al., 2019).

The gut microbiota engages in complex interactions with the host, likely affecting muscle metabolism, growth, and atrophy through multiple pathways, ultimately influencing muscle quality and function (Lahiri et al., 2019; Yan et al., 2023).

Imbalances in the gut microbiota may negatively impact muscle health by initiating systemic inflammatory responses (Mendes et al., 2023; Agostini et al., 2023). Research suggests that disruptions in gut microbial balance can compromise intestinal barrier integrity, increase intestinal permeability, and facilitate the translocation of bacterial endotoxins, such as lipopolysaccharides, into the bloodstream, thereby promoting systemic inflammation (Chen et al., 2024; Li et al., 2024). This chronic, low-grade inflammatory state is recognized as a key pathological mechanism underlying sarcopenia, where inflammatory mediators like TNF-α and IL-6, activated through the NF-κB signalling pathway, inhibit muscle protein synthesis and accelerate muscle protein breakdown (Bian et al., 2017; Xuekel et al., 2024).

Moreover, metabolites produced by the gut microbiota, including short-chain fatty acids (SCFAs) and branched-chain amino acids (BCAAs), play critical roles in regulating muscle metabolism and function. SCFAs such as butyrate and propionate exhibit anti-inflammatory and immunomodulatory properties, and enhance muscle protein synthesis through activation of the AMP-activated protein kinase (AMPK) signalling pathway (den Besten et al., 2013). Moreover, butyric acid, inhibits histone deacetylases, resulting in increased histone acetylation and subsequent changes in gene expression (de Conti et al., 2013). This epigenetic regulation can influence the differentiation and regenerative capacity of muscle cells, ultimately affecting muscle health and function (Ticinesi et al., 2024). Reduced fecal butyrate levels have been observed in older individuals with low muscle mass (Yuan, 2024). When gut microbial balance is disrupted, the production of these beneficial metabolites declines, adversely affecting muscle health (Yuan, 2024). Additionally, elevated levels of harmful metabolites, such as indole and p-cresol, have been linked to muscle atrophy (Kang et al., 2024; Xu et al., 2024). Furthermore, the gut microbiota can modulate host gene expression by influencing the expression of microRNAs (miRNAs). Research has shown that shifts in gut microbiota composition are associated with altered expression patterns of certain miRNAs, which may play key roles in regulating muscle metabolism and processes linked to muscle atrophy. Transplanting gut microbiota from healthy individuals into mice with muscle wasting has been shown to significantly improve muscle mass and function, highlighting the therapeutic potential of gut microbiota restoration for treating muscle wasting. Moreover, supplementation with specific probiotics and prebiotics has been reported to enhance muscle mass and function in patients with sarcopenia, further reinforcing the critical role of the gut microbiota in the development and management of sarcopenia (Qaisar et al., 2024; Nistor-Cseppento et al., 2022).

### 3.10 MicroRNA

miRNAs are a class of non-coding RNA molecules that regulate gene expression by either inhibiting translation or promoting the degradation of specific mRNA targets (Dong et al., 2021). Non-coding RNAs, particularly microRNAs (miRNAs) and long non-coding RNAs (lncRNAs), have emerged as crucial regulators of muscle atrophy and regeneration. These molecules influence muscle mass and function by modulating multiple signalling pathways, including the insulin-like growth factor 1 (IGF-1)/AKT/mTOR and the TGF-β/SMAD pathway (Sohi and Dilworth, 2015; Pinheiro and Naya, 2021).

Circulating levels of many microRNAs (c-miRNAs) have identified as potential biomarkers for sarcopenia.

It has been demonstrated that the upregulation of miR-141-3p in ovariectomized mice contributes to mitochondrial dysfunction by inhibiting FKBP prolyl isomerase 5 (FKBP5) and Fibin (Lee et al., 2021). Lower levels of miR-133b and miR-206 have been associated with sarcopenia, often linked to malnutrition in older individuals (Iannone et al., 2020). The downregulation of miR-532-3p, an inflammation-associated miRNA, regulates the apoptotic pathway during sarcopenia progression by targeting BCL2 antagonist/killer 1 (BAK1) (Chen et al., 2020). A significant downregulation of miR-29b has been observed in elderly individuals with sarcopenia, particularly those with cardiovascular risk factors such as diabetes, hypertension, and dyslipidemia (He et al., 2022). Plasma levels of miR-21, along with miR-206, have been identified as indicators of accelerated sarcopenia (Qaisar et al., 2021). Upregulation of miR-126-5p has demonstrated high diagnostic accuracy for sarcopenia (Faraldi et al., 2024), while miR-146a upregulation contributes to sarcopenia by modulating the IRAK1/TRAF6/NF-κB signaling pathway (Jin et al., 2023). miR-133b, miR-206, miR-155, miR-208b, miR-222, miR-210, miR-328, and miR-499 downregulation has been associated with sarcopenia (Iannone et al., 2020; He et al., 2020; Salamanna et al., 2023).

Also, miR-532-3p downregulation has been associated with sarcopenia progression though inflammation mediated via BAK1 regulation (Chen et al., 2020). On the contrary the downregulation of miR-1290 promotes myoblast differentiation and protects against myotube atrophy through activation of the Akt/p70/FoxO3 signalling pathway (Che et al., 2021). Also, miR-672-5p downregulation has been shown to alleviate symptoms of sarcopenia (Ahmad et al., 2019).

### 3.11 The myostatin pathway

Myostatin/activin, key components of the TGF-β superfamily, plays a fundamental role in controlling muscle mass and function. Some studies suggest increased myostatin levels with age (Wilhelmsen et al., 2024) and indeed myostatin/activin pathway dysregulation is strongly implicated in the development of sarcopenia (Amthor and Hoogaars, 2012). Secreted mainly by muscle cells, myostatin and activins exert both autocrine and paracrine effects by interacting with Activin type IIB receptors (ActRIIB) on muscle cell membranes (Li J. et al., 2021). This receptor activation subsequently recruits type I receptors such as ALK4 or ALK5, triggering the phosphorylation of SMAD2 and SMAD3. These phosphorylated SMAD proteins form a complex with SMAD4 (Welle, 2009), which then migrates into the nucleus to regulate gene transcription. Through this mechanism, the pathway downregulates key myogenic factors necessary for muscle growth and differentiation, while also suppressing protein synthesis (Armaghani and Han, 2020). In addition to inhibiting anabolic processes, myostatin/activin signaling enhances muscle protein degradation via the ubiquitin-proteasome system and may also promote autophagy pathways, collectively accelerating muscle wasting (Sartori et al., 2009).

### 3.12 Novelty and innovation

Myonuclear alterations, mitochondrial dysfunction, disrupted protein homeostasis, muscle fibers denervation, fibro-adipogenic progenitor depletion, and microvascular dysfunction all occur simultaneously in the ageing muscle. We review novel evidence on the cellular crosstalk among the key players of the sarcopenia, which is the consequence of these processes, emphasizing the role of fibroblastic-like cells, adipocytes, and locally recruited immune cells in shaping the aging muscle microenvironment and the molecular hints to hierarchy among them. By integrating cellular, molecular, and vascular perspectives, this work sets the stage for novel intervention strategies aimed at preserving muscle health in aging populations.

## 4 Conclusion and future perspective

The future of research into muscle aging holds promising potential for addressing the challenges of sarcopenia. Advances in molecular biology, particularly in the understanding of satellite cells and myonuclear domain regulation, may pave the way for therapies that restore muscle regenerative capacity and mitigate muscle degeneration. Personalized exercise regimens and nutritional strategies, tailored to individual genetic and metabolic profiles, are likely to become more prevalent. As we further unravel the complexities of muscle aging, a more proactive approach to healthy aging may emerge, allowing individuals to maintain muscle health, reduce the risk of frailty, and enjoy an improved quality of life in later years.
